# Unsuspected Dengue and Acute Febrile Illness in Rural and Semi-Urban Southern Sri Lanka

**DOI:** 10.3201/eid1802.110962

**Published:** 2012-02

**Authors:** Megan E. Reller, Champika Bodinayake, Ajith Nagahawatte, Vasantha Devasiri, Wasantha Kodikara-Arachichi, John J. Strouse, Anne Broadwater, Truls Østbye, Aravinda de Silva, Christopher W. Woods

**Affiliations:** Johns Hopkins University School of Medicine, Baltimore, Maryland, USA (M.E. Reller, J.J. Strouse);; Medical Faculty of University of Ruhuna, Galle, Sri Lanka (C. Bodinayake, A. Nagahawatte, V. Devasiri, W. Kodikara-Arachichi);; University of North Carolina School of Medicine, Chapel Hill, North Carolina, USA (A. Broadwater, A. de Silva);; Duke University School of Medicine, Durham, North Carolina, USA (T. Østbye, C.W. Woods)

**Keywords:** dengue, dengue virus, viruses, acute dengue, secondary dengue, recurrent dengue, acute febrile illness, young adults, pandemic, rural, semi-urban, Sri Lanka. dengue hemorrhagic fever, vector-borne diseases, mosquitoes, *Aedes aegypti*

## Abstract

Dengue virus (DENV), a globally emerging cause of undifferentiated fever, has been documented in the heavily urbanized western coast of Sri Lanka since the 1960s. New areas of Sri Lanka are now being affected, and the reported number and severity of cases have increased. To study emerging DENV in southern Sri Lanka, we obtained epidemiologic and clinical data and acute- and convalescent-phase serum samples from patients >2 years old with febrile illness. We tested paired serum samples for DENV IgG and IgM and serotyped virus by using isolation and reverse transcription PCR. We identified acute DENV infection (serotypes 2, 3, and 4) in 54 (6.3%) of 859 patients. Only 14% of patients had clinically suspected dengue; however, 54% had serologically confirmed acute or past DENV infection. DENV is a major and largely unrecognized cause of fever in southern Sri Lanka, especially in young adults.

## Abstract 2

Acute dengue may be under-recognized in other regions because of limited studies and tools for rapid diagnosis.

Dengue virus (DENV), with 4 antigenically distinct serotypes (DEN1–4), is the most common cause of arboviral disease; ≈50 million cases of dengue occur annually in >100 countries (*1*). Manifestations of dengue infection range from asymptomatic or mild febrile illness to circulatory failure and death from dengue hemorrhagic fever (DHF). Urbanization and geographic expansion of the primary vector for DENV, *Aedes aegypti*, have fueled the current global dengue pandemic (*2*).

DENV has been documented in the Indian subcontinent since the 1960s, and there are recent reports of DENV in new areas and of severe disease (*2**,**3*). Epidemic dengue was first recognized in Colombo, the capital of Sri Lanka, in 1965–1966 (*4*). Outpatient, clinic-based surveillance at Colombo’s Lady Ridgeway Children’s Hospital during 1980–1984 found dengue accounted for 16% of acute febrile illness, among which 66% were secondary (recurrent) dengue cases. A 1980–1985 school-based study found a baseline DENV seroprevalence of 50% in Colombo and a 6-month dengue incidence of 15.6%, of which 37% were secondary cases (*4*). In the early 1980s, severe dengue was rare in Sri Lanka: <10 reported cases were DHF (*4*). However, since 1989, many cases of DHF have been reported from the heavily urbanized western coastal belt of Sri Lanka, which includes Colombo (*5*), and cases have recently been reported elsewhere in the country.

DENV began emerging in southern Sri Lanka recently. To define the epidemiology of dengue in this previously unstudied region, we prospectively enrolled patients seeking care for acute febrile illness at a local hospital. Study participants lived in Galle, a seaport city (population 100,000), and the surrounding coastal plain and heavily vegetated foothills at the southernmost tip of the island nation. During the study months, the temperature ranged from highs of 27.5°C to 32°C and lows of 24°C to 26°C, and rainfall was variable (mean 301 mm, range 36–657 mm).

## Materials and Methods

### Febrile Cohort

During March–October 2007, we recruited patients in the emergency department, acute care clinics, and adult and pediatric wards of Teaching Hospital Karapitiya in Galle, the largest (1,300-bed) hospital in southern Sri Lanka. Consecutive febrile (>38°C tympanic) patients >2 years old without trauma or hospitalization within 7 days were eligible for study participation. Study doctors verified eligibility and willingness to return for convalescent-phase follow-up and obtained written consent from patients or from parents (for patients <18 years old) and assent from those >12–17 years old. The institutional review boards of the University of Ruhuna, Johns Hopkins University, and Duke University Medical Center approved the study.

Study personnel recorded epidemiologic and clinical data on a standardized form. Study doctors obtained blood for on-site, clinician-requested testing and off-site, research-related testing. Patients returned for clinical and serologic follow-up 2–4 weeks later or were visited in their home if unable to return and their address was known. Blood and serum samples were stored promptly at −80°C and shipped on dry ice to the University of North Carolina School of Medicine (laboratory of A. de S.), where paired serum samples were tested by ELISA and acute-phase serum samples were cultured and tested by PCR.

### Serologic Testing for Dengue

#### IgM ELISA

We performed dengue IgM capture ELISA as described (*6*), except we used anti-flavivirus monoclonal antibody (mAb) 4G2 followed by enzyme-conjugated goat anti-mouse IgG to detect captured DENV antigen. In brief, 96-well plates were coated (overnight, 4°C) with 100 μL/well (1 ng/μL) of goat anti-human IgM (Sigma, St. Louis, MO, USA) at a concentration of 0.1 mol/L in carbonate buffer (pH 9.6). Plates were washed 3× in Tris-buffered saline with 0.2% Tween 20 (TBST) and blocked with 200 μL/well of 1× Tris-buffered saline with 0.05% Tween 20 and 3% nonfat dry milk. Paired serum samples were tested on the same plate. Diluted serum (1:50) was loaded in duplicate and incubated (37°C, 1 h) to capture IgM antibody. Unbound antibody was washed, and wells were successively incubated with DENV antigen (mix of serotypes DEN1–4), mouse anti-flavivirus 4G2 mAb, and human-absorbed alkaline phosphatase (AP)–conjugated goat anti-mouse IgG antibody (Sigma). Optical density (OD) was measured at 405 nm after final incubation with AP substrate.

#### IgG ELISA

Dengue IgG ELISA was performed as described (*7*). Plates were coated overnight (4°C) with 100 μL/well of mouse anti-flavivirus 4G2 mAb at a concentration of 0.1 mol/L in carbonate buffer (pH 9.6) and then washed 3× in TBST. Plates were then blocked with standard diluents and successively incubated (37°C, 1 h) with DEN1–4 antigen, diluted serum (1:100) in duplicate wells, and AP-conjugated goat anti-human IgG (Fc portion), with 3 washings (TBST) between incubations. Plates were read at 405 nm after a final incubation with AP substrate (15 min, room temperature, in the dark).

### Serologic Interpretation

We defined acute dengue as IgG seroconversion (acute-phase OD <0.20 and convalescent-phase OD >0.20) or as a substantial increase in antibody titer (convalescent-phase IgG OD >0.30 or IgM OD >0.20 than acute-phase). Acute primary (first episode) and acute secondary (recurrent) dengue were distinguished by the absence or presence of IgG (OD <0.35 and >0.35, respectively) in acute-phase serum samples. The presence of IgG without a substantial increase in titer defined past dengue infection. Seroprevalence was defined as the presence of IgG (OD >0.20) in acute-phase serum samples; other samples were seronegative. For each ELISA, we used 2 negative control human serum samples, each tested in duplicate. Positive cut-off values were determined during assay validation and were based on the mean OD +2 SD for negative control serum specimens.

### Isolation of Dengue

Individual 15-μL aliquots of undiluted acute-phase serum (from all patients with confirmed acute dengue as well as negative and positive controls) were each mixed with 185 μL of Eagle minimal essential medium supplemented with 2% fetal bovine serum, and then each was added to C6/36 cells (27°C, 5% CO_2_). After 7 and 10 days, cells were fixed and a direct immunofluorescent antibody assay was performed by using anti-dengue mAb (2H2-Alexa488) (*8*). Positive samples were centrifuged (1,500 × *g*, 5 min). Supernatants containing virus were supplemented with 20% fetal bovine serum, divided into aliquots, and preserved at −80°C.

### Dengue Virus Neutralization

We tested the convalescent-phase serum samples from 35 patients with serologically defined acute dengue. Dengue neutralizing antibodies were detected by using a flow cytometry–based assay as described (*9*). In brief, serially diluted immune serum samples were incubated with each of the 4 dengue serotypes (37°C, 1 h). Next, the virus and serum mixture was added to a human monocyte cell line (U937DC-SIGN) that was engineered to express the dengue receptor DC-SIGN and incubated with 5% CO_2_ (37°C, 24 h). The cells were washed, fixed, and stained with Alexa 488–conjugated anti-dengue mAb 2H2. The percentage of infected cells was measured by flow cytometry. GraphPad Prism 4 for Windows (www.graphpad.com/welcome.htm) and nonlinear regression analysis were used to calculate 50% neutralization values.

### PCR for DENV

To yield cDNA, we used RNA eluted serum samples (QIAmp Viral RNA Mini Kit; QIAGEN, Valencia, CA, USA) from 25–140 µL of acute-phase serum samples, reverse transcriptase (RT), and DENV downstream consensus primer D2 or random primers. To confirm and serotype DENV, we used consensus primers D1 and D2 to amplify cDNA by standard PCR; positive first-round PCR products were diluted 1:100 and used as template in second-round PCR with consensus primer D1 and nested serotype DEN1–4 type-specific primers (*10*). Negative controls were included for the RT and PCR steps. The negative control for the RT step consisted of a sample to which all reagents, except RNA, were added. The negative control step for PCR consisted of a sample to which all reagents, except cDNA, were added.

### Statistical Analysis

Proportions were compared by the χ^2^ test or Fisher exact test and continuous variables by Student *t* test or the rank sum test, if not normally distributed. Analyses were performed by using Stata IC 11.0 (StataCorp LP, College Station, TX, USA).

## Results

### Febrile Cohort

Paired serum samples to identify acute and past dengue were available for 859 (79.6%) of 1,079 patients enrolled. The likelihood of follow-up did not differ by age (p = 0.30), sex (p = 0.22), or level of education (p = 0.74). Most (90.2%) patients reported rural residence, and follow-up was more likely among rural (80.6%) than urban (69.5%) dwellers (p = 0.008). The reported duration of fever and illness was similar in patients who did and did not return for follow-up.

Among the 859 patients with paired serum samples, 61.2% were male, and the median age was 30.7 years (interquartile range [IQR] 19–48 years), which did not differ by sex (p = 0.97). The median reported duration of fever and of illness before seeking medical care was 3 days (IQR 2–5 and 2–7 days, respectively). Many patients (36.3%) reported having taken an antimicrobial drug for the illness. The median time between acute-phase and convalescent-phase follow-up was 21 days (IQR 15–32 days).

### Acute Dengue

**Figure 1:**
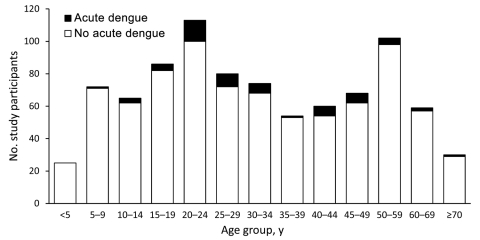
Age distribution of patients with and without acute dengue, southern Sri Lanka, 2007.

Acute dengue occurred during each month of the study and accounted for 3.0% (May) to 11.1% (October) of acute febrile illnesses. Fifty-four patients (6.3%) had acute dengue (27 primary and 27 secondary infections). Of patients >18 and <18 years old, 48 (7.0%) and 6 (3.5%), respectively, had acute dengue (p = 0.09). The age distribution of patients with and without acute dengue is shown in Figure 1. The median age of patients with acute dengue was 27.6 years (IQR 22–45 days), and a similar proportion were male versus female (p = 0.26).

The clinical features of patients with and without acute dengue are detailed in Table 1. Headache was the most frequent (75.9%) symptom, and lethargy and muscle and joint pain were also reported by >50% of patients with dengue; however, these symptoms were just as frequent in patients without dengue. Patients with dengue were less likely than those without it to report cough and sore throat and to have lymphadenopathy, but they were more likely to have conjunctivitis. Although gastrointestinal symptoms and signs were uncommon overall, diarrhea, jaundice, hepatomegaly, and abdominal tenderness occurred more frequently in dengue patients. Patients with acute dengue had statistically significantly lower leukocyte and platelet counts than patients without dengue. No dengue patients had petechiae.

**Table 1:** Clinical characteristics of febrile patients with and without acute dengue, southern Sri Lanka, 2007*

Characteristic	Acute dengue, n = 54	Not acute dengue, n = 805	p value
Symptoms			
Headache	75.9	78.1	0.70
Sore throat	13.0	29.3	0.01
Cough	35.2	58.5	0.001
Dyspnea	15.1	16.7	0.76
Joint pain	50.9	44.3	0.35
Muscle pain	57.7	48.7	0.21
Lethargy	68.6	66.6	0.76
Abdominal pain	25.0	19.0	0.29
Emesis	43.4	36.9	0.35
Diarrhea	22.2	10.7	0.01
Dysuria	16.7	14.4	0.65
Oliguria	14.8	9.1	0.17
Signs			
Mean temperature, °C, ± SD	38.5 ± 0.70	38.4 ± 0.61	0.66
Mean heart rate, beats/min, ± SD	85 ± 17	84 ± 16	0.57
Mean systolic blood pressure, mm Hg, ± SD	114 ± 16	111 ± 16	0.32
Mean diastolic blood pressure, mm Hg, ± SD	72 ± 16	73 ± 11	0.58
Conjunctivitis	27.8	14.0	0.006
Pharyngeal erythema or exudates	5.6	14.0	0.08
Lymphadenopathy	3.7	24	0.001
Jaundice	7.6	1.4	0.001
Lung crackles	5.7	14.2	0.08
Tender abdomen on palpation	18.5	9.4	0.03
Hepatomegaly	11.3	5.0	0.05
Swollen joint	1.9	0.5	0.27
Rash	5.6	2.3	0.14
Laboratory parameters			
Leukocytes/μL, median (IQR)	5,300 (3,600–12,000)	8,000 (5,800–11,100)	0.009
ANC/μL, median (IQR)	3,600 (2,304–8,560)	5,396 (3,534–7,980)	0.03
ALC/μL, median (IQR)	1,602 (1,044–2,400)	2,085 (1,540–2,829)	0.002
Hemoglobin, g/dL, mean ± SD	12.9 ± 1.6	12.6 ± 1.7	0.25
Platelets, × 1,000/μL, mean (IQR)	190 (156–242)	232 (190–293)	0.0002

*Values are percentages unless otherwise stated. Leukocytes, white blood cell count; ANC, absolute neutrophil count; ALC, absolute lymphocyte count; IQR, interquartile range.

Only 3 acute dengue patients (men 23–27 years old; 2 with secondary and 1 with primary infection) had platelet counts consistent with DHF (<100,000/μL) (*11*). At admission, 2 of the 3 men reported myalgia and arthralgia; all 3 had headache, leukopenia (leukocytes 2,400–3,600 cells/μL), severe thrombocytopenia (platelets 19,000–47,000/μL), and compensated shock (blood pressure 100–110/70–80 mm Hg, with heart rate 88–100 beats/min). Additional laboratory testing only included determination of transaminase levels, which were elevated in the 1 patient in whom they were evaluated. All 3 patients were well at follow-up.

Patients with acute dengue were more likely than those with other causes of fever to be hospitalized (92.6% vs. 71.3%; p = 0.001), but their duration of stay was similar (median 5 vs. 4 days [IQR 3–7 and 3–6 days, respectively]; p = 0.23). A presumptive clinical diagnosis was available for 791 patients, including 50 with acute dengue. Few adults and children were suspected to have acute dengue on the basis of clinical features (3.3% vs. 1.2%, respectively; p = 0.16), and only 7 of the 23 patients (all adults) with clinically suspected dengue had laboratory-confirmed infection (positive predictive value 30.4%, 95% CI 27.2–33.6). Clinical diagnosis also had poor sensitivity (14.0%; 95% CI 11.6–16.4) and high specificity (97.8%; 95% CI 96.8–98.9). Compared with other patients, those with suspected dengue were more likely to be admitted to the hospital (70.4% vs. 95.7%; p = 0.008) and less likely to be treated with antimicrobial drugs (72.6% vs. 20.0%; p = 0.02). However, 50.0% of those with confirmed acute dengue reported taking antimicrobial drugs before seeking medical care.

Clinical illness was similar in patients with primary and secondary dengue. Median platelet counts were 186,000/μL (IQR 153,000–234,000/μL) and 198,000/μL (IQR 160,000–270,000/μL) for patients with primary and secondary dengue, respectively (p = 0.60). Diarrhea was more common in patients with secondary than with primary dengue (33.3% vs. 11.1%; p = 0.05), as were jaundice (14.8% vs. 0.0%; p = 0.04) and hepatomegaly (22.2 vs. 0.0%; p = 0.01). The duration of illness before patients sought care was similar for those with primary and secondary dengue. Patients with primary dengue were as likely as those with secondary dengue to be admitted to the hospital (both 92.6%; p = 1.0) and to remain for a similar duration (median 5 days for both [IQR 3–6 and 3–8 days, respectively]; p = 0.89).

### Acute or Past Dengue

**Figure 2:**
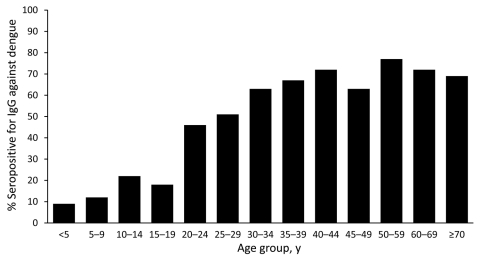
Presence of IgG against dengue in febrile patients, by age, southern Sri Lanka, 2007.

Fifty-four percent (464/859) of the study cohort had either acute or past dengue (see Table 2 for patient characteristics). The overall seroprevalence was 50.9% (437/859 patients) because the 27 patients with acute primary infections were seronegative at enrollment. The proportion of patients who were seropositive at enrollment increased in each older age group, from 9% in those <5 years old to 72% in those 40–44 years old (Figure 2). Overall, seropositivity was more likely in male than in female patients (55.9% vs. 42.9%; p<0.0001), and in urban than in rural dwellers (75.3% vs. 52.0%; p<0.0001).

**Table 2:** Demographic characteristics of febrile patients with or without evidence of dengue virus infection, southern Sri Lanka, 2007*

Characteristic	Acute dengue, n = 54†	Past dengue, n = 410†	Not dengue, n = 395†	p value
Median age, years (IQR)	27.6 (22.4–44.5)	41.3 (27.0–53.4)	21.7 (13.3–35.3)	0.0001
Male	68.5	66.3	54.9	0.002
Residence				0.001
Rural	86.8	88.2	95.4	
Urban	13.2	11.8	4.6	
Type of work				<0.001
Home	11.5	25.9	27.8	
Laborer	19.2	32.4	18.3	
Farmer, e.g., rice paddy	5.8	3.0	2.3	
Merchant	3.9	4.0	2.1	
Student	13.5	7.3	37.8	
Other	46.2	27.4	11.8	
Swim/bathe/wade in freshwater				0.63
None	74.1	73.1	76.4	
River	20.4	13.5	12.2	
Paddy field	3.7	10.8	9.1	
Pond/lake	0	1.0	1.0	
Other	1.9	1.7	1.3	
Water source				0.003
Tap	42.6	35.1	25.5	
Boiled	3.7	8.8	10.2	
Well	51.9	54.9	64.4	
Other	1.9	1.2	0	

*Except for median age, data are percentages. IQR, interquartile range. †Values are percentages unless otherwise stated.

### Confirmation of Acute Dengue and Serotyping of Virus

We isolated DENV from 12 (22.2%) of the 54 acute dengue patients. The likelihood of isolating DENV was higher when the patient had fewer reported days of fever (median 3 vs. 4 days; p = 0.04), and DENV was isolated almost exclusively from patients with primary versus secondary dengue (91.7% vs. 8.3%; p = 0.001).

Dengue PCR results were positive in 19 (35.2%) patients; positive results were higher among patients with positive versus negative culture results (75.0% vs. 23.8%; p = 0.001). Fever duration was similar in patients with dengue-positive and -negative PCR results (median 4 days; p = 0.22). A positive PCR result was not statistically more likely in patients with primary versus those with secondary dengue (57.9% vs. 42.1%; p = 0.39). PCR testing confirmed serotype DEN2 (2 primary cases), D3 (8 primary, 7 secondary), and D4 (1 primary, 1 secondary); no cases of serotype DEN1 were identified. DENV neutralization testing confirmed the specific presence of dengue antibodies in 33 (94.3%) of 35 IgG-positive serum samples (95% CI 80.8–99.3).

## Discussion

We sought to define the epidemiology of dengue in the southern tip of Sri Lanka. We prospectively enrolled patients with a reproducible criterion (documented fever) and rigorously distinguished acute from past infections by using paired serum samples. The seroprevalence of dengue in our cohort increased with age; however, half of those 20–25 years old were seronegative. In contrast, 50% of children in Colombo are positive for dengue IgG antibody by 5 years of age, and >70% have been found to be seropositive by 12 years of age (*4**,**12*). The high proportion of susceptible adults in Galle suggests the recent emergence of DENV in this area (*4**,**5*).

Dengue is considered an urban and peri-urban disease, and the current pandemic is partly attributed to increasingly populous cities (*2*). We documented cases of acute dengue during March–October in rural and semi-urban areas. In rural Cambodia, dengue is a major cause of hospitalization and death among children, with delays in recognition and care-seeking contributing to its impact in rural areas (*13**,**14*). Discarded water storage jars and concrete water tanks are commonly used breeding sites for the vector (*15*), and mosquito populations may be higher in rural than urban areas (*16*). Dengue has also been documented in rural Vietnam (*17*). Control efforts in urban Sri Lanka have included covering cement water tanks, which are established breeding sites for *Aedes* spp (*18*); however, data to direct control efforts in Southern Sri Lanka are lacking.

We describe the clinical manifestations of endemic dengue in an unselected febrile cohort in southern Sri Lanka. Dengue accounted for 6.3% (54/859) of acute febrile illnesses in this cohort; serotypes DEN2–4 caused illness, with DEN3 being the most frequent. In the cohort (predominantly adults who sought care early in illness), manifestations of acute dengue were similar to those of other causes of fever, except for more diarrhea, jaundice, and abdominal tenderness and less cough and sore throat. Ramos et al. (*19*) similarly identified the presence of diarrhea and absence of respiratory symptoms as early features of confirmed acute dengue infection in adults; however, 53.5% of the patients in that study were excluded from analyses because only single serum samples were tested.

We identified a disease spectrum different from that of classic severe dengue reported in hospital-based studies during epidemics in urban centers (Colombo and Kandy) in Sri Lanka (*20**–**22*); the spectrum we report is similar to that described in studies of acute febrile illness elsewhere in Asia. In a report from Singapore, headache, retro-orbital pain, arthralgia, anorexia, nausea, and vomiting were symptoms statistically associated with dengue, but, as in our study, the symptoms were common in their entire febrile cohort (*23*). In the same study, signs more suggestive of dengue were infrequent: red eyes (33.6% of patients), rash (11.2%), and bleeding (7.5%). The only signs we found statistically associated with acute dengue were jaundice, abdominal tenderness, and hepatomegaly. Others have found abnormal test results for liver function to be common among patients with secondary dengue (*24*). That we observed jaundice only in secondary cases lends credence to the hypothesis that host response to the virus may be a major factor in the pathogenesis of dengue (*24*).

Our finding that only 14% of patients with dengue were identified clinically is consistent with the recent emergence of dengue in southern Sri Lanka. In addition, the finding emphasizes the difficulties with clinical diagnosis, particularly in unselected patients with recent onset of fever (median 3 days) in the absence of a recognized epidemic. Clinical acumen is difficult to develop when confirmatory testing is not available, even in a subset of patients. This fact highlights the need for rapid, accurate point-of-care diagnosis (*25*), which could also limit the frequent use of unnecessary antimicrobial drugs.

A limitation of many studies is that diagnosis of acute dengue was made on the basis of a single serum sample. In studies of severe dengue in urban Sri Lanka (*20**,**22*) and in a more recent case-control study of dengue (*26*), acute dengue was defined by a positive result from a PanBio dual IgM/IgG rapid strip test (PanBio Pty Ltd., Brisbane, Australia) (*27*), which was performed on a single serum sample obtained on day 7 of illness. This strategy may be adequate during an epidemic in a patient with classic severe dengue; however, in a setting of high dengue seroprevalence and lower pretest probability (undifferentiated fever, no recognized epidemic), a single positive serologic test result may more often denote past dengue, and the true cause of illness might remain unrecognized and untreated.

Unique strengths of our study include rigorous confirmation of acute infection by World Health Organization criteria (*28*), a large sample size with 80% follow-up (critical to reference-standard diagnosis and assessment of outcomes), and prospective clinical correlation. To confirm dengue, the World Health Organization requires detection of virus by isolation or PCR or a 4-fold rise in antibody in paired serum samples. Cases with supportive serologic test results (single high antibody titer) are considered probable cases because antibody may not be present early (poor sensitivity) or may represent past infection (poor specificity) (*28*). In our study, we would have identified only secondary cases if a probable case definition had been used because IgG is initially absent in primary dengue. We assessed paired serum samples by using an ELISA comparable to the more difficult and time-consuming hemagglutinin inhibition test used for determining dengue seroprevalence in populations (*7*) and the ELISA used for dengue surveillance in Colombo, Sri Lanka (*12*). A recent systematic review scrutinized clinical studies designed to aid clinicians in resource-poor settings by identifying features predictive of acute dengue (*29*). Most studies had myriad methodologic flaws, including inadequate diagnosis, which made prediction impossible. We prospectively studied unselected patients by using rigorous diagnostic criteria to minimize recall, selection, and diagnostic verification bias.

A limitation of serologic testing is that serologic cross-reactions occur between dengue and other flaviviruses, but we believe this is unlikely to bias our results. West Nile virus has not been described in the region, yellow fever is not present in Asia, and Japanese encephalitis is of low endemicity, with no recent outbreaks reported in the area. Furthermore, we used dengue virus neutralization testing to confirm the specific presence of dengue in a subset of IgG-positive serum samples in addition to performing PCR and viral isolation on the acute-phase serum samples of patients with serologically confirmed acute dengue. The median duration of fever in those in whom virus was isolated is consistent with the reported persistence of viremia for 5–6 days after onset of symptoms (*1**,**30*). Because most patients were adults, we could not compare the clinical features of symptomatic dengue in adults versus those in children.

We have not delineated the full clinical spectrum of dengue, which would require a prospective population-based study. However, we believe that the population studied is representative of patients in the region with clinically noteworthy symptomatic dengue: Teaching Hospital Karapitiya has a large catchment area and is 1 of 2 public teaching hospitals in the Southern Province, and there are no large private hospitals in the area. Patients with fulminant dengue may die before hospital evaluation, but most, including indigent patients from outlying areas, seek care because of free access.

In summary, we found that dengue is responsible for 6.3% of undifferentiated febrile illnesses in southern Sri Lanka. We identified relatively mild disease, despite identifying mostly serotype DEN3 infection, which is difficult to distinguish from other acute febrile illnesses, and we found acute infections were mostly in young adults. We hypothesize that the burden and spectrum of acute dengue is under-recognized in other regions because of limited study and limited tools for rapid diagnosis. Clinical recognition is poor for less severe dengue, particularly, perhaps, when endemicity is low or transmission is sporadic. Assays to measure antibody in single serum samples may be misleading, especially early in illness, and PCR and viral isolation are often unavailable and may be insensitive. Other causes of acute febrile illness require antimicrobial drug therapies, but dengue only requires supportive care; thus, improved low-cost diagnostic tools are urgently needed to guide clinical management.
